# A systematic review of the quality of distal radius systematic reviews: Methodology and reporting assessment

**DOI:** 10.1371/journal.pone.0206895

**Published:** 2019-01-23

**Authors:** João Carlos Belloti, Aldo Okamura, Jordana Scheeren, Flávio Faloppa, Vinícius Ynoe de Moraes

**Affiliations:** 1 Department of Orthopedics and Traumatology, Division of Hand Surgery, Universidade Federal de São Paulo, Sao Paulo, Brazil; 2 Grupo cirurgia da mão e microcirurgia, Hospital Alvorada Moema, São Paulo, São Paulo, Brazil; University of Mississippi Medical Center, UNITED STATES

## Abstract

**Background:**

Many systematic reviews (SRs) have been published about the various treatments for distal radius fractures (DRF). The heterogeneity of SRs results may come from the misuse of SR methods, and literature overviews have demonstrated that SRs should be considered with caution as they may not always be synonymous with high-quality standards. Our objective is to evaluate the quality of published SRs on the treatment of DRF through these tools.

**Methods:**

The methods utilized in this review were previously published in the PROSPERO database. We considered SRs of surgical and nonsurgical interventions for acute DRF in adults. A comprehensive search strategy was performed in the MEDLINE database (inception to May 2017) and we manually searched the grey literature for non-indexed research. Data were independently extracted by two authors. We assessed SR internal validity and reporting using AMSTAR (Assessing the Methodological Quality of Systematic Reviews and PRISMA (Preferred Reporting Items for Systematic Reviews and Meta-Analyzes). Scores were calculated as the sum of reported items. We also extracted article characteristics and provided Spearman’s correlation measurements.

**Results:**

Forty-one articles fulfilled the eligibility criteria. The mean score for PRISMA was 15.90 (CI 95%, 13.9–17.89) and AMSTAR was 6.48 (CI 95% 5.72–7.23). SRs that considered only RCTs had better AMSTAR [7.56 (2.1) vs. 5.62 (2.3); p = 0.014] and PRISMA scores [18.61 (5.22) vs. 13.93 (6.47), p = 0.027]. The presence of meta-analysis on the SRs altered PRISMA scores [19.17 (4.75) vs. 10.21 (4.51), p = 0.001] and AMSTAR scores [7.68 (1.9) vs. 4.39 (1.66), p = 0.001]. Journal impact factor or declaration of conflict of interest did not change PRISMA and AMSTAR scores. We found substantial inter observer agreement for PRISMA (0.82, 95% CI 0.62–0.94; p = 0.01) and AMSTAR (0.65, 95% CI 0.43–0.81; p = 0.01), and moderate correlation between PRISMA and AMSTAR scores (0.83, 95% CI 0.62–0.92; p = 0.01).

**Conclusions:**

DRF RCT-only SRs have better PRISMA and AMSTAR scores. These tools have substantial inter-observer agreement and moderate inter-tool correlation. We exposed the current research panorama and pointed out some factors that can contribute to improvements on the topic.

## Introduction

Distal radius fractures (DRF) are frequent and afflict both the young and older population. It is a topic of prolific research [1]. Randomized controlled trials have attempted to scrutinize the best methods for treating DRF, derived from methods of conservative treatment to advanced strategies of plate osteossynthesis. DRF impacts the health system due to the effect on a young labor force and also in the elderly, and as such, surgeons, researchers and policymakers have pursued RCTs, with relevant support from governmental agencies, independent researchers and industry.

The increasing number of RCTs on the topic has created a need to organize the data, as well as summarize the generated evidence. In an ideal scenario, systematic reviews (SRs) should have driven efforts toward better quality information [2]. However, SRs are sometimes misleading and may result in conflicting results, even when considering the same population and condition [3]. Frequent deceptive situations are related to the inclusion of studies other than RCTs, meta-analysis conducted without consideration of unexplained heterogeneity, and the lack of outcome-focused analysis [4].

Within the scope of DRF treatments, a great number of SRs have been conducted, and thus there is a need to appraise their quality and internal validity [5]. The appraisal of relevant available research is of value for pinpointing strengths and weakness on the topic. We have delineated this study based on the hypothesis that a majority of DRFs SRs lack quality, and may be responsible from the conflicting results on the subject. The aim of the study is threefold: (1) describe the state of art of SRs on DRF treatment; (2) assess study quality (internal validity and reporting) and measure correlation with various aspects of SRs (SR methods and number of words); and (3) correlation measurements for PRISMA and AMSTAR scores.

## Materials and methods

The methods from this review were previously published in the PROSPERO database [6], under number CRD42017070212 (http://www.crd.york.ac.uk/PROSPERO/display_record.asp?ID=CRD42017070212), showed in S1 PROSPERO Protocol. A local committee provided ethical consent under number CAAE 76473517.6.0000.5505.

### Literature search

From inception to May 2017 a comprehensive literature search was conducted in Medline, with no language restrictions. The search strategy was performed using two methods:

Method 1- Utilizing the terms (with the boolean term OR): “distal radius fracture”, “Colles’ fracture”, "wrist fracture” and study design terms (with the boolean term OR): “systematic review”, “review”, meta-analysis, metanalysis. Distal radius search terms and study design search terms were combined with the AND boolean term.
Method 2- from PUBMED clinical query tool (https://www.ncbi.nlm.nih.gov/pubmed/clinical) utilizing “distal radius fracture”. This feature includes one pre-defined filter for systematic reviews.

Both search results were analyzed independently by 2 researchers (J.S, V.Y.M), discrepancies were solved by the aid of the senior author (J.C.B). We chose MEDLINE as the only assessed database as it is available for a worldwide audience and it includes most relevant research.

### Inclusion criteria

Systematic reviews (with or without meta-analysis) that included any studies (RCTs and non-RCTs) that assessed DRFs treatment (operative and non-operative) in an adult population.

### Exclusion criteria

Narrative reviews or diagnosis or risk-assessment (case-controls, cohorts) SRs were excluded. All so-called SRs that lacked a transparent literature search and strategy for their data approach were considered as narrative and excluded from our analysis. Diagnostic and anesthetic interventions were also excluded.

### Methodology (internal validity) assessment and quality reporting

Data derived from all assessed papers were considered for the elaboration of a descriptive table that presents some of the SR evidence (and characteristics) on the topic. We obtained data: Conflict of Interest declaration status, Country of origin, type of treatment, total Sum of Patients, PRISMA statement ciation, number of Words and types of study designs included in the SRs.

AMSTAR (A Measurement Tool to Assess Systematic Reviews) [7] was applied in in order to assess the methodological quality of systematic reviews. This is a validated tool that encompasses eleven dichotomous queries relevant to the internal validity of systematic reviews. Queries are related to: study design (Q1); search and study inclusion/exclusion (Q2-5), study characteristics (Q6), SRs internal vality (Q7-10), conflict of interest (Q11). AMSTAR has maximum 11 points score, higher scores indicates better quality (Appendix 1).

PRISMA (Preferred Reporting Items for Systematic Reviews and Meta-Analyses) [8] is a tool that aids in analysis of the reporting of systematic reviews and meta-analysis. It considers 27 items. For this analysis, we considered all 27 items and considered the sum of positive answers as the final score with higher scores indicating better reporting quality (Appendix 2).

Study data acquisition, AMSTAR and PRISMA assessment were performed in duplicate. In terms of comparison, we have *a priori* defined some subgroups for a comparative analysis of SR quality: (1) grouping according to impact factor (FI<1.5 vs. FI>1.5); (2) presence of associated meta-analysis (yes/no); (3) RCTs only versus other than RCTs SRs; (4) declaration of interest (yes/no); and (5) length of the article in words.

### Data analysis

Data was verified for normality by visual judgment in addition to Shapiro-wilk test. We demonstrated data as descriptive and provided means and standard deviation when applicable (AMSTAR and PRISMA Scores). For non-normally distributed data, we have inputed medians and interquartile range (IQR). Inter-observer agreement and score correlation were considered with a Spearman correlation and the following classification: more than 0.8, perfect agreement; 0.61–0.8, substantial agreement, 0.60–0.41, moderate agreement and below 0.4 indicates low agreement. For inferential statistics analysis, we considered the AMSTAR and PRISMA scores. Means were compared by unpaired Student T-test and medians with Mann-Whitney U test. We considered as significant when p<0.05.

## Results

**Fig 1:**
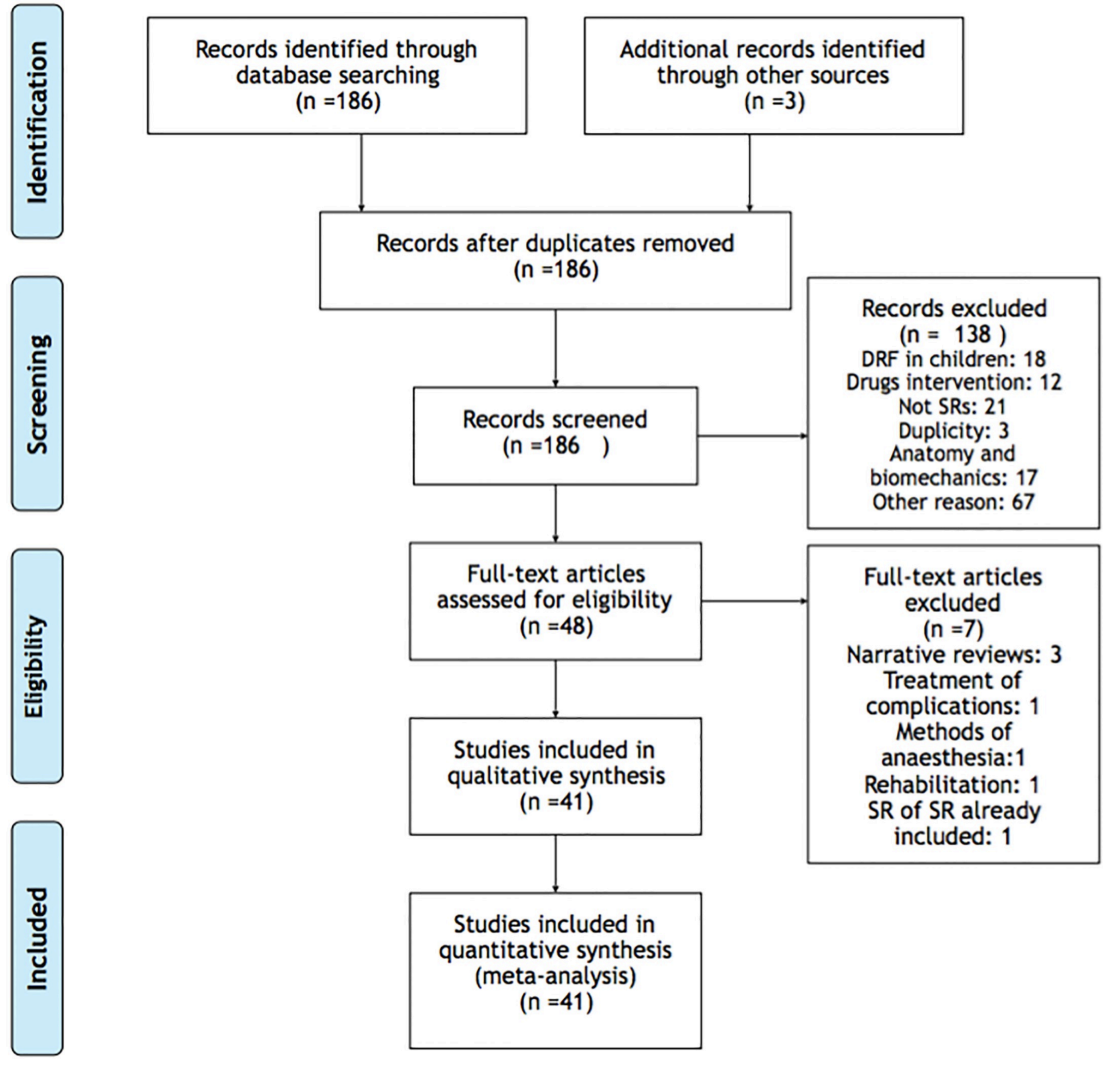
Flow diagram.

From 186 studies, we have excluded 138 after title and abstract assessment. Forty-one studies were included in the final assessment. The PRISMA flowchart, including reasoning for study exclusions are diagrammed in Fig 1. We have detailed study characteristics in Table 1. Overall quantitative data is provided in Table 2 [9–49]. Seven systematic reviews were excluded after full-text assessment for the following reasons: three Cochrane reviews: one about methods of anaesthesia in DRFs [50], one about rehabilitation after DR treatment [51] and one SR of studies already included [52]; three narrative reviews with no specific scope on treatment [53–55]; and one that considered DRFs complications treatment [56].

**Table 1:** Characteristics of included studies.

Author, year	Declared Conflit of Interest	Country	Treatment	Total Sum of Patients	PRISMA Statement cited	Number of Words	Study Design
**Asadollahi, 2013** [9]	Yes	Australia	ORIF (locked vs. non locking plates)	47	No	4715	case series
**Azzi, 2016** [10]	Yes	Canada	ORIF (dorsal vs. volar plates)	6278	Yes	5877	RCTs, prospective and retrospective series
**Baradaran, 2016** [11]	Yes	Iran	Styloid fracture fixation	1340	Yes	1409	N/A
**Bentohami, 2013** [12]	Yes	Netherlands	ORIF (volar plates)	1817	Yes	5748	RCTs, prospective and retrospective series
**Chen, 2016** [13]	Yes	China	Operative, Nonsurgical	883	No	4342	RCTs, prospective and retrospective series
**Cui, 2011** [14]	No	China	EF, ORIF	738	Yes	5538	RCTs
**Cui, 2012** [15]	Yes	China	EF (dynamic vs. static)	998	No	5874	RCTs
**Diaz-Garcia, 2011** [16]	No	USA	VLPs, Non-BrEF, BrEF, KW, CI	N/A	No	1304	N/A
**Esposito, 2013** [17]	Yes	Canada	EF, ORIF	707	No	6193	RCTs
**Farrar, 2008** [18]	No	UK	Dorsal vs. Radial cast	N/A	No	748	N/A
**Franceschi, 2015** [19]	Yes	Italy	ORIF (volar plates), KW	1306	Yes	8631	RCTs, prospective series
**Handoll, 2007** [20]	Yes	UK	KW, Conservative		Yes	32284	
**Handoll, 2008** [21]	Yes	UK	Reduction, Anesthesia	404	Yes	14668	RCTs
**Handoll, 2008** [22]	Yes	UK	Plaster, Brace	4215	Yes	44953	RCTs
**Handoll, 2010** [23]	Yes	UK	Bone grafts	874	Yes	37239	RCTs
**Harman, 2015** [24]	Yes	Canada	ORIF (volar plates), KW	875	Yes	6869	RCTs
**Hoang-Kim, 2009** [25]	No	Italy	EF	433	No	4502	RCTs
**Jordan, 2015** [26]	Yes	UK	IMN, VLP, CAST, EF	380	Yes	5822	RCTs, biomechanics, case series (prospective, retrospective)
**Ju, 2015** [27]	Yes	China	Operative, Nonsurgical	889	Yes	5743	RCTs, prospective and retrospective series
**Margaliot, 2005** [28]	Yes	USA	EF, ORIF	1520	No	10994	RCTs, prospective and retrospective series
**Modi, 2016** [29]	Yes	UK	EF (dynamic vs. static)	1151	No	5173	RCTs, case series (prospective, retrospective)
**Mulders, 2017** [30]	Yes	Netherlands	pronator quadratus repair, no repair	169	Yes	4824	
**Paksima, 2004** [31]	No	USA	CI, EF, ORIF. KW, OREF	N/A	No	4595	RCTs, prospective and retrospective series
**Qiu, 2015** [32]	Yes	China	Surgical treatment (complications)	1805	No	6121	RCTs
**Suhm, 2008** [33]	No	UK	Bone graft (with or without)	580	No	4938	retrospective, biomechanics
**Trevisan, 2013** [34]	Yes	Italy	Operative, Nonsurgical		No	845	
**Valdes, 2014** [35]	No	China	PT (Oriented vs. Supervised)	381	No	6846	RCTs
**Walenkamp, 2013** [36]	Yes	Netherlands	EF, ORIF (volar plates)	174	No	5670	RCTs
**Wan Li, 2016** [37]	No	China	EF (Bridging vs. non bridging)	905	No	5339	N/A
**Wang, 2012** [38]	Yes	China	EF, ORIF	824	No	5947	RCTs, prospective and retrospective series
**Wang, 2016** [39]	Yes	China	IMN, ORIF (volar plates)	369	Yes	5369	RCTs
**Wei, 2012** [40]	No	Canada	EF, ORIF	1011	No	790	RCTs, prospective and retrospective series
**Wei, 2013** [41]	Yes	China	ORIF (dorsal vs. volar plates)	952	No	3361	RCTs, prospective and retrospective series
**Wijffels, 2014** [42]	Yes	Netherlands	Ulnar styloid (union, nonunion)	365	Yes	6007	Observational studies
**Xie, 2013** [43]	Yes	China	EF, ORIF	760	Yes	4772	RCTs
**Xu, 2015** [44]	Yes	China	BrEF, Non- BrEF, conservative, ORIF (dorsal, volar, dorsal and volar plates)	1805	No	5153	RCTs
**Yu, 2016** [45]	Yes	China	ORIF, Conservative	653	No	5522	RCTs
**Zhang, 2014** [46]	Yes	China	ORIF (volar plates), EF	445	Yes	5500	RCTs
**Zhang, 2016** [47]	No	China	EF, ORIF		Yes	6240	RCTs
**Zhang, 2017** [48]	Yes	China	IMN, ORIF (volar plates)	463	No	5166	RCTs, prospective series
**Zong, 2015** [49]	Yes	China	ORIF (volar plates), KW	875	Yes	4943	RCTs

EF, external fixation; ORIF, open reduction and internal fixation; RCT, randomized clinical trial; CI, cast immobilization; KW, kirschner wire; OREF, open reduction and external fixation; Br EF, bridging external fixation; Non-Br EF, non-bridging external fixation; VLP, volar locking plate; IMN, intramedullary nailling; PT, physical therapy.

**Table 2:** Quantitative data.

	Menas or Median*	95% Confidence interval or IQR**
PRISMA E1	15.1	13.28–16.91
PRISMA E2	16.8	14.49–19.11
PRISMA MEAN	15.90	13.9–17.89
AMSTAR E1	6.36	5.46–7.26
AMSTAR E2	6.94	6.1–7.7
AMSTAR MEAN	6.48	5.72–7.23
NUMEBER OF WORDS	5522*	4743–6157
NUMBER OF PATIENTS	874*	422–1151

E1: Examiner 1; E2: Examiner 2;

* Median

**IQR: interquartile range

We found substantial inter-rater correlation for PRISMA (Spearman correlation, 0.82, 95% CI 0.62–0.94; p = 0.01) and AMSTAR (Spearman correlation, 0.65, 95% CI 0.43–0.81; p = 0.01). We found moderate inter-tool correlation between PRISMA and AMSTAR scores (Spearman correlation, 0.83, 95% CI 0.62–0.92; p = 0.01).

When comparing SR that considered only RCTs (22 SRs) versus SR that were nonRCTs (19 SRs), we found differences in AMSTAR scores [7.56 (2.1) vs. 5.62 (2.3); Mann-Whitney U test, p = 0.014] and PRISMA scores [18.61 (5.22) vs. 13.93 (6.47); Mann-Whitney U test, p = 0.027, respectively].

When comparing SR that declared conflict of interest (31 SRs) versus SR with no information on the topic (10 SRs), we found no differences in AMSTAR scores [5.16 (2.27) vs. 6.98 (2.31); Mann-Whitney U test, p = 0.06]. However, differences were found for PRISMA scores [17.61 (5.50) vs. 11.30 (6.5); Mann-Whitney U test, p = 0.017].

Journal impact factor seems did not influence the quality of the SRs (Low IF, 34 SRs High IF, 7 SRs): PRISMA scores [15.47 (6.11) vs. 18.00 (7.29); Mann-Whitney U test, p = 0.46] and AMSTAR scores [(6.34 (2.28) vs. 7.14 (3.00); Mann-Whitney U test, p = 0.55)].

The presence (27 SRs) or not (14 SRs) of meta-analysis on the SRs altered PRISMA scores [19.17 (4.75) vs. 10.21 (4.51) vs. Mann-Whitney U test, p = 0.001] and AMSTAR scores [7.68 (1.9) vs. 4.39 (1.66); Mann-Whitney U test, p = 0.001].

## Discussion

This study evaluated the quality of systematic reviews published in the literature regarding the treatment of distal radius fractures in adults. Well-conducted systematic reviews are the gold standard for summarizing evidence for treatment decisions. However, systematic reviews are not always synonymous with high-quality evidence, since misused methodology may lead to bias, just as with any other type of study. To evaluate the quality of systemic reviews, our study analysis utilized PRISMA as a guideline for how a meta-analysis should be reported, and AMSTAR, which specifically focuses on adequate review methodology. Adie et al [57] were the first to assess meta-analyses in the surgery setting with the PRISMA statement and provided the standards for assessing what is known about a particular topic.

Our analysis of the 41 systematic reviews on treatment of distal radius fracture in adults showed that, on average, 6.48 of the 11 items (59%) in AMSTAR were adequately reported, as well as 15.9 of the 27 items (59%) in PRISMA. There was substantial inter observer agreement for PRISMA (Spearman correlation, 0.82, 95% CI 0.62–0.94; p = 0.01) and AMSTAR (Spearman correlation, 0.65, 95% CI 0.43–0.81; p = 0.01), as well as moderate agreement between PRISMA and AMSTAR scores (Spearman correlation, 0.83, 95% CI 0.62–0.92; p = 0.01). Therefore, we believe that both questionnaires show good applicability and represent useful methods of assessment regarding the quality of systematic reviews. It is important to note that PRISMA was not initially designed for methodological assessments, and was intended only as a guide/checklist for reporting.

In our study, AMSTAR and PRISMA scores were better in systematic reviews that considered exclusively randomized controlled trials. Of the 41 studies evaluated, 22 included only RCTs. Similar to the PRISMA statement, the Consolidated Standards of Reporting Trials (CONSORT) [58] offers a standard way for authors to prepare reports about trial findings, facilitating their complete and transparent reporting, reducing the influence of bias on their results, and aiding their critical appraisal and interpretation. In this way, RCTs tend to present a better methodology and, consequently, systematic reviews that include only RCTs, also tend to have better scores for PRISMA and AMSTAR.

In general, AMSTAR and PRISMA scores were better in SRs with meta-analyses, compared to systematic reviews without meta-analysis. The PRISMA statement evolved from the earlier Quality of Reporting of Meta-analyzes (QUORUM) [59] collaboration checklist, whose objective was to improve the quality of reports of meta-analyzes of RCTs. Meta-analyses are useful tools for summarizing surgical evidence, as they can sum multiple data on a particular research question, but they may also be prone to methodological biases if not well conducted. Studies of low methodological quality may alter the interpretation of the benefit of the intervention [60].

There was no evidence of association between the journal impact factor and the quality of the systematic reviews evaluated in our study. Some studies show that even when systematic reviews were published in high-impact journals, endorsement of PRISMA in the instructions for authors was not a guarantee of compliance [61]. In our study, Cochrane reviews gave scores for PRISMA that were approximately 10 points higher and AMSTAR 3 points higher than other evaluated systematic reviews. Cochrane reviews follow strict guidelines and protocols and have been consistently superior to the methodology than other studies [62–64].

We found no differences in AMSTAR scores, when comparing SR that declared conflict of interest (31 SRs) versus SR with no information on the topic (10 SRs). However, differences were found for PRISMA scores [17.61 (5.50) vs. 11.30 (6.5); Mann-Whitney U test, p = 0.017]]. Report clearly possible sources of funding or support refers to the last item of both questionnaires. Cullis et al. [65] found that PRISMA item 27 was adequately reported in 26% of studies, whereas in our study we found this item reported in 76%.

The methods utilized in this review were previously published in the PROSPERO database. A pre-determined protocol is important because it may restrict the opportunities for biased *post hoc* changes in methodology [66]. Thus, the prior publication represents a positive aspect of this work and adds greater credibility.

We must consider ways to improve the methodological quality of systematic reviews and meta-analyzes on the treatment of distal radius fractures. Ideally, more journals should approve, or at least insist that the authors follow PRISMA. Tao et al. [67] evaluated 146 leading medical journals about the use of the PRISMA Statement, and it was referred to in the instructions to authors for 27% (40/146) of journals. For now, only the Cochrane Database of Systematic Reviews and PLOS ONE officially endorse PRISMA, as well as the Annals of Surgery, BJU International, BMJ Open, The International Journal of Surgery and the Journal of Trauma and Acute Care Surgery.

We found a moderate agreement between the length of the article in words and AMSTAR and PRISMA scores. Adie et al [57] also found the same positive association between manuscript length and PRISMA and AMSTAR statements, even as Biondi-Zoccai et al [68] found an association between the length of the article and quality of reporting of meta-analyses (QUOROM) score. However, this finding is not confirmed by more recent analyses [67]. Constraints on space and limits of the number of words imposed by journals might influence on the quality of the systematic reviews. Our findings suggest that manuscripts can achieve optimal quality scores if sufficient space is provided by the journal.

### Limitations

Our review has its limitations. We attempted to identify all the systematic reviews and meta-analyzes already published regarding the treatment of distal radius fracture in adults in an electronic database. We tried to minimize our limitations by having two authors perform the screening, selection and extraction independently.

Our scoring systems were binary (YES or NO) for evaluation of AMSTAR and PRISMA items, similar to Adie et al. [57]. McGee et al. [69] used a scaled score system, accommodating the criteria in which adequacy was partially achieved. We evaluated the studies simply, which may be a limitation of this study. A concern is related to the fact that most of the comparative analysis may be overlapping, as some primary studies are a source of data for multiple SRs. This issue should be addressed by another specific research piece on the topic.

The lack of studies similar to ours, whose objective is to evaluate the methodology of the systematic reviews, provides us with little for comparison. Thus, we used the few studies on this topic, which also include interventions in other areas of medicine.

## Conclusions

Published systematic reviews on the treatment of distal radius fractures in adults present methodological flaws, just half of the studies included only RCTs and about 35% of RVs had no metanalysis. DRF RCT-only SRs and SRs with meta-analysis have better PRISMA and AMSTAR scores. These tools have substantial inter-observer agreement and moderate inter-tool correlation. Greater adherence with PRISMA and AMSTAR would produce better quality studies, with a positive impact on medical knowledge about adult DRF treatment.

## Supporting information

S1 File PROSPERO protocol. (PDF)

S2 File PRISMA checklist. (PDF)
