# Supplementary material for: A systematic review of the quality of distal radius systematic reviews: Methodology and reporting assessment
Source: PLoS One. 2019 Jan 23;14(1):e0206895. doi: 10.1371/journal.pone.0206895 (PMC6343870; doi:10.1371/journal.pone.0206895)
Supplement: S1 File — (PDF) [file pone.0206895.s001.pdf]

PROSPERO International prospective register of systematic reviews

Quality assessment of distal radius systematic reviews

Vincius Ynoe Moraes, João Carlos Belloti, Jordana Schreen

Citation

Vincius Ynoe Moraes, João Carlos Belloti, Jordana Schreen. Quality assessment of distal radius systematic reviews. PROSPERO 2017:CRD42017070212 Available from http://www.crd.york.ac.uk/PROSPERO_REBRANDING/display_record.asp?ID=CRD42017070212

Review question(s)

1.What is the quality of distal radius systematic reviews?

2. Are they well reported?

3. How confident can one be regarding the internal validity of the results?

Searches

PubMed will be searched. In addition, PROSPERO database will be searched in an attempt to identify ongoing research.

English publications only.

Types of study to be included

Systematic reviews (of randomized or quasi randomized clinical trials) that included adult patients treated from distal radius fractures.

Condition or domain being studied

Adults presenting distal radius fractures.

Participants/ population

Systematic reviews (of randomized or quasi randomized clinical trials) that included adult patients treated (any active treatment) for distal radius fractures.

Intervention(s), exposure(s)

Any surgical or conservative method of treatment.

Comparator(s)/ control

Any surgical or conservative method of treatment.

Outcome(s)

Primary outcomes

Methodological quality:

AMSTAR (Shea et al. BMC Med Res Methodol 15; 7:10, 2007).

Secondary outcomes

Quality of reporting:PRISMA (Galvão et al. Epidemiol Serv Saude. 2015).

Data extraction, (selection and coding)

Two authors (VY and JC) will search for eligible studies and also will plot bibliometrics for the included studies.

Risk of bias (quality) assessment

Bias assessed by AMSTAR (Shea et al. BMC Med Res Methodol 15; 7:10, 2007).

Strategy for data synthesis

Data will be synthesized as qualitative (exposed as plain text and tables)

Two authors (VY e JC) will apply: AMSTAR, ROBIS and PRISMA.

Agreement (Pearson/Spearman) will be assessed for the tools.

AMSTAR and PRISMA tools will be demonstrated, and means and SDs.

Analysis of subgroups or subsets

Groups of studies will be analyzed by inferential statistics whether they differ:

Journal's impact factor (FI <1,5 vs, FI>1,5)

Declaration of conflict of interest (yes/no)

Number of institutions involved (single vs. multicentre)

Industry funding (yes/no)

Number of words.

Dissemination plans

Publishing in an indexed journal.

Contact details for further information

Vincius Ynoe de Moraes

Rua Borges Lagoa, 778 So Paulo - SP - Brazil

vymoraes@gmail.com

Organisational affiliation of the review

UNIFESP/EPM

www.unifesp.br

Review team

Dr Vincius Ynoe Moraes, UNIFESP/EPM
Professor João Carlos Belloti, UNIFESP/EPM
Dr Jordana Schreen, UNIFESP/EPM

Anticipated or actual start date

04 July 2017

Anticipated completion date

15 September 2017

Funding sources/sponsors

None

Conflicts of interest

None known

Language
English

Country
Brazil

Subject index terms status
Subject indexing assigned by CRD

Subject index terms
Humans; Radius; Research Design; Wrist Joint

Stage of review
Ongoing

Date of registration in PROSPERO
13 July 2017

Date of publication of this revision
13 July 2017

Stage of review at time of this submission	Started	Completed
Preliminary searches	Yes	Yes
Piloting of the study selection process	Yes	Yes
Formal screening of search results against eligibility criteria	Yes	No
Data extraction	No	No
Risk of bias (quality) assessment	No	No
Data analysis	No	No

PROSPERO
International prospective register of systematic reviews
The information in this record has been provided by the named contact for this review. CRD has accepted this information in good faith and registered the review in PROSPERO. CRD bears no responsibility or liability for the content of this registration record, any associated files or external websites.
